# Clinical Strategies for Complete Denture Rehabilitation in a Patient with Parkinson Disease and Reduced Neuromuscular Control

**DOI:** 10.1155/2015/352878

**Published:** 2015-02-08

**Authors:** Satheesh B. Haralur

**Affiliations:** Department of Prosthodontics, College of Dentistry, King Khalid University, Abha 61417, Saudi Arabia

## Abstract

The dentist has a large role in geriatric health care for the ever increasing elder population with associated physical and neurological disorders. The Parkinson disease is progressive neurological disorder with resting tremor, bradykinesia, akinesia, and postural instability. The psychological components of disease include depression, anxiety, and cognitive deficiency. Poor oral hygiene, increased susceptibility for dental caries, and periodontal diseases predispose them to early edentulism. The number of Parkinson affected patients visiting dental clinic seeking complete denture is growing. This case report explains the steps involved in the complete denture rehabilitation of Parkinson patient. The effective prosthesis will help in alleviating functional, aesthetic, and psychological disabilities of the patient.

## 1. Introduction

The prevalence of Parkinson's disease (PD) is on the rise from last few decades due to the improved life expectancy across the world. The PD is second most common neurodegeneration diseases after Alzheimer disease [1]. It is genetically linked disease, with the mean age of onset around 57 years, and a slight predilection for men. The PD has four cardinal signs of resting tremor, bradykinesia, akinesia, and postural instability. The orofacial findings of these patients include mask-like face due to akinesia, impaired speech, xerostomia, and dysphagia with saliva drooling from the corner of mouth [2]. Although PD patients drool, they actually produce less saliva than normal age matched controls, thus increasing their risk of decay, leading to an increased need, perhaps, for dentures. The patient gait is slow with forward flexion of upper body. The oral hygiene is severely compromised due to lack of muscular control, depression [3], and cognitive problems [4]. The PD patients due to poor hygiene are prone to increased periodontal diseases, dental caries, and early loss of teeth. The well-fitting denture significantly contributes to the eating and social integration [5]. The successful complete denture rehabilitation largely depends on the ability of the patient to control the denture with oral musculature. The denture retention and control in PD patients is further compromised due to thick ropey saliva, xerostomia, and rigid muscles. The psychological component like depression, cognitive problems, and apathy further jeopardise the successful fabrication and utilisation of complete denture. The anticholinergic drugs, sometimes used for tremor or for bladder overactivity, produce dry mouth, which needs to be considered in designing any oral intervention. The treatment plan strategy should consider both physical and psychological challenges associated with the disease. The patient management should be compassionate and caring, and it should include effective time management [6]. This case report describes the complete denture rehabilitation strategy and procedure involved in a patient with Parkinson's disease.

## 2. Case Report

**Figure 1:**
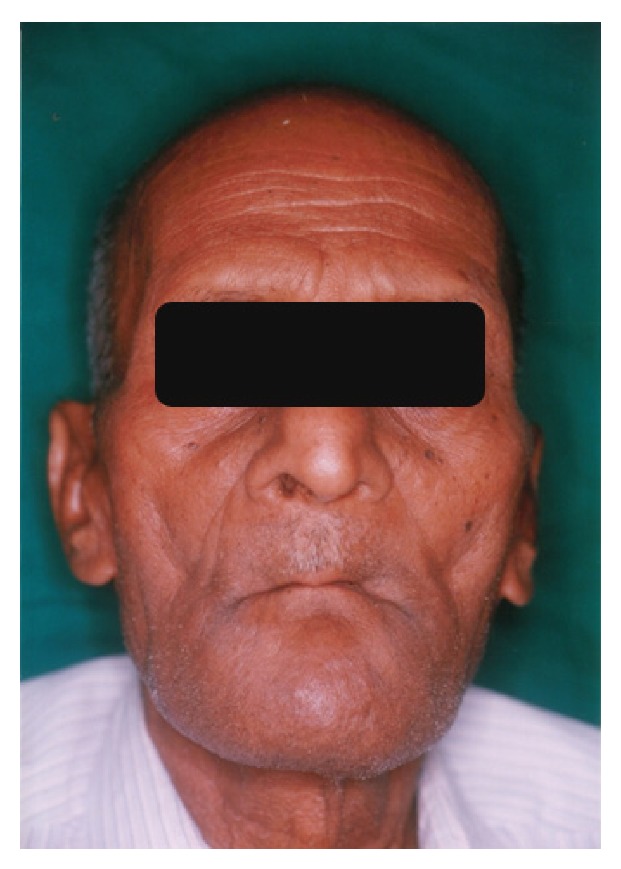
Completely edentulous Parkinson patient with mask-like face and fixed gaze.

**Figure 2:**
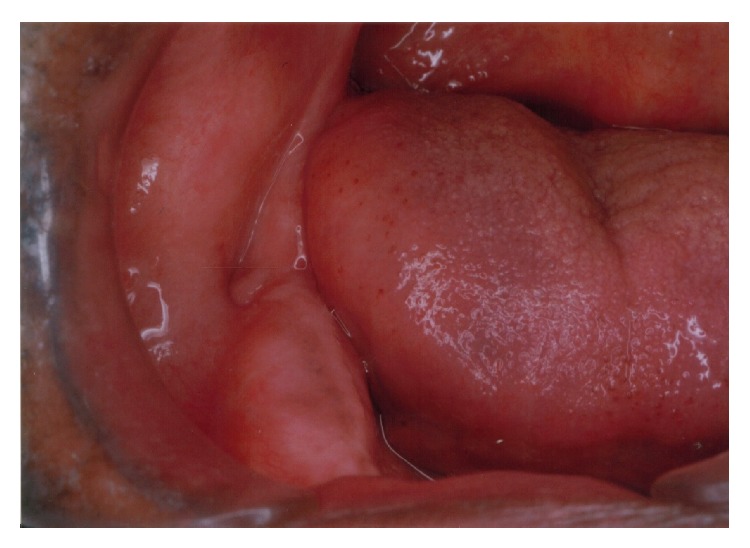
Postsurgical depression due to dentigerous cyst enucleation at third molar region.

A 65-year-old completely edentulous male patient with Parkinson's disease was referred to prosthodontic clinic for complete denture rehabilitation (Figure 1). He was diagnosed with PD at 61 years of age; since then he was under Levodopa medication. Patient presented the history of surgical enucleation for dentigerous cyst at right side mandible ten years ago. After surgery, the patient complained of slight paraesthesia of lip on the operated side. The patient presented with festinated gait and forward flexion of the body. He required the assistance to walk and get in/out of the dental chair indicating a lower level of muscle coordination. The speech of the patient was soft, hurried, and monotonous. The extraoral examination showed the lack of facial expression and reduced blinking of eyes. The mandibular movement showed the slight trembling and limited movement of the lips during conversation. The patient was depressive and showed cognitive dysfunction. The intraoral examination revealed the reduced salivary flow, with postsurgical depression (0.5 cm × 0.5 cm diameter) in the region of lower right third molar (Figure 2). Both maxillary and mandibular residual ridges were high and well-rounded, with deep palatal vault and Type III soft-hard palate junction. The slight difficulty in swallowing was noticed in the patient due to the initial stage of bradykinesia. Temporomandibular joint examination showed no significant pathological deviation. The OPG radiograph was made to rule out the recurrence of dentigerous cyst. The patients consulting neurophysician opinion was sought regarding the fitness and required precaution during dental treatment. The treatment objective was the complete denture rehabilitation of the patient to improve his nutrition status through improved masticatory efficiency. The objective also included the improvement in speech and enhancing his psychological status. The treatment options were discussed with the patient and his wife. The advantage of an implant retained prosthesis was explained; due to socioeconomic constraints of the patient the conventional completed denture treatment plan was finalized. The treatment plan was explained to the patient, and informed consent was obtained for the treatment plan.

The patient was advised to consume the PD medicine (Levodopa) one hour prior to treatment. The appointments were fixed in the morning time for the short duration of 45 minutes. The patient was requested to empty his urinary bladder before treatment initiation to avoid urinary urgency and incontinence. A special emphasis was made to treat the patient in compassionate, caring environment to alleviate anxiety and frustration behaviour. The patient's wife was made to sit next to the patient to reduce the anxiety and to help in interpreting the patient's speech. The sympathetic approach was followed to allow slow response rate of patient during communication.

**Figure 3:**
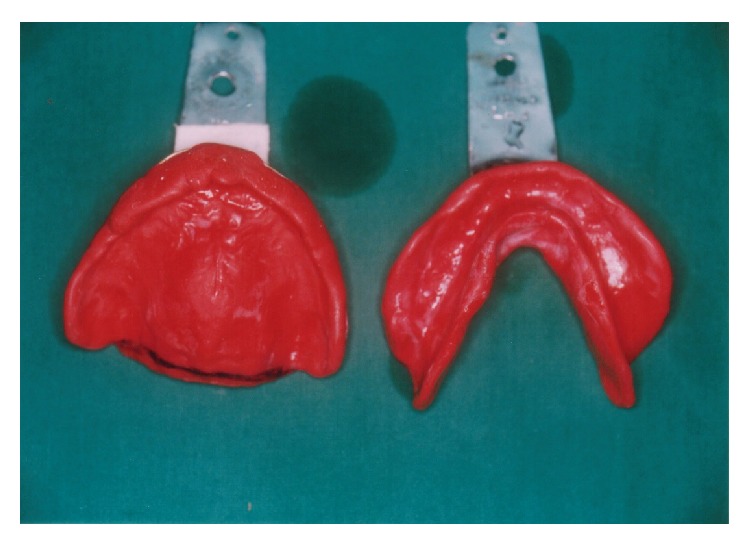
Primary impression in impression compound.

**Figure 4:**
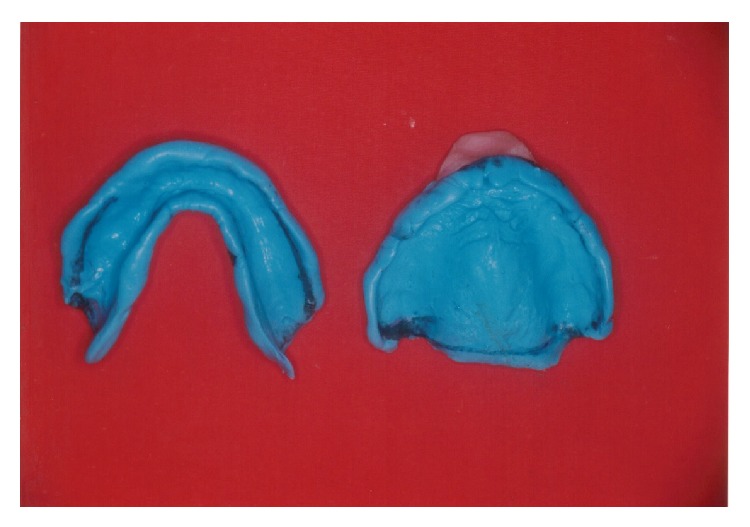
Secondary impression with border moulding.

**Figure 5:**
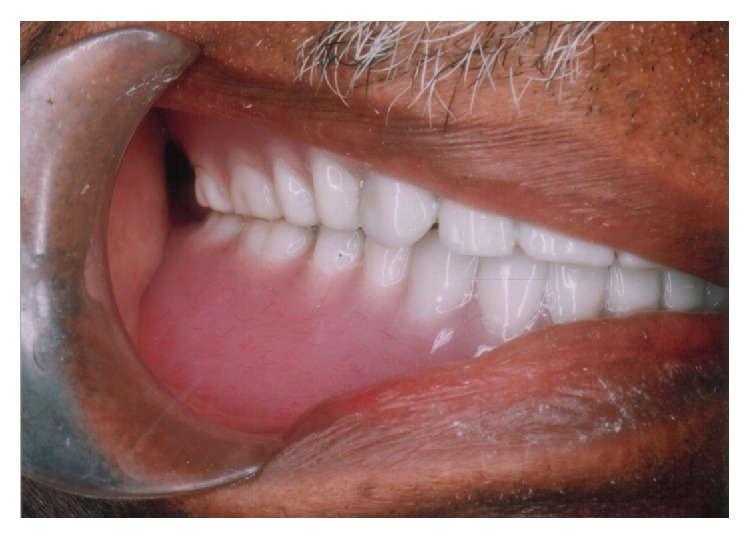
Posterior teeth at occlusion.

**Figure 6:**
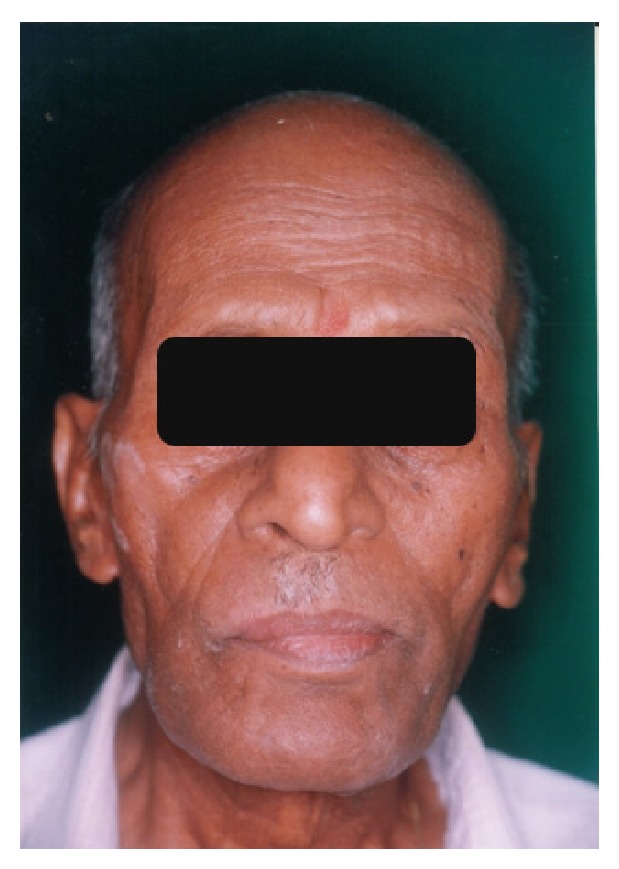
Patient after complete denture rehabilitation.

The dental chair was inclined at 45 degrees to facilitate the swallowing except during jaw relation procedure. Since the patient was unable to open the mouth for a long time the primary impression was made from impression compound (Figure 3). It provided the dual advantage of quick setting and subsequent correction. The special tray was fabricated from autopolymerizing acrylic for selective pressure impression method. The border moulding was done again in an incremental step from modelling plastic impression compound. Light body additional silicone impression material was used for final secondary impression (Figure 4). The vertical height of occlusion was set slightly less than the normal. The jaw movement exercises were explained and demonstrated to the patient and his wife. The patient was requested to practice the mandible movement at home prior to jaw relation appointment. The maxilla mandibular horizontal jaw relation was established by bilateral manipulation technique. The Alu-wax was used for recording jaw relation. Another set of similar record blocks was made from modelling plastic impression compound to record neutral zone. The softened compound was placed inside the patient's mouth, and he was requested to perform physiological muscle functions like sucking, swallowing, and phonetics. The procedure was repeated by softening compound due to poor muscular activity of the patient. The split putty index was made from the contoured impression compound to guide the technician in arranging the teeth in the neutral zone. The denture teeth were arranged in lingualized occlusion. The aesthetic try-in and jaw relation verification was done on a trial denture. The selective grinding procedure was performed on the articulator and inside the patient mouth. The occlusal correction was performed over two appointments to eliminate the occlusal interferences (Figure 5). The denture was fabricated by high impact denture base resin (Figure 6). The soft liner relining was done at corresponding surgical defect area. The patient was advised to frequently sip water. After consultation with his neurophysician, the carboxymethylcellulose artificial salivary substitute was advised. Though the denture was adequately retentive, the water based denture adhesive (Fixodent) was prescribed to enhance the patient's denture confidence. The denture cleansing tablets were also prescribed to improve the denture hygiene. The importance of prosthesis hygiene and removal of the prosthesis during sleep was emphasized to the patient. The follow-up recall visits were programmed for continuous evaluation and required correction of denture.

## 3. Discussion

Geriatric health care is a critical part of health care systems around the world due to the rapidly increasing elderly population. Dentist plays an important role in geriatric health care and can contribute significantly in restoring the quality of life in elderly patients [7]. Several diseases of the aged population are neurological disorders. The Parkinson disease (PD) is a progressive neurological disorder that affects numerous motor and nonmotor functions. The motor symptoms of PD include resting tremors, involuntary movements, facial and limb rigidity, bradykinesia, and akathisia. The major nonmotor symptoms include drooling, swallowing difficulties, xerostomia, bladder dysfunction, depression, anxiety, cognitive impairment, and orthostatic hypotension [4, 8, 9]. These symptoms complicate the dental treatment plans, execution, and outcome. These disabilities must be taken into consideration during treatment for the favourable prognosis.

The compassionate, caring approach with PD patient is important to overcome the anxiety and better treatment compliance from the patient. Cognitive impairment, dementia, and difficulty in verbal communication should be handled sympathetically. Hence, it is advised for the dentist to introduce himself every appointment. The stress is known to exacerbate the tremor and uncontrolled movement during treatment. The smiling, direct eye contact, and gentle touch are known to alleviate the anxiety [9]. The caregiver's presence beside the patient is also helpful for patient confidence and for interpreting the patient's speech. The short, mid-morning appointments are ideal for the patient. The tremors are less in morning and drug is most effective 60–90 minutes after its intake [2, 10]. The communication with the patient is improved by using closed-ended questions and allowing adequate time for the patient to respond. Effective communication is important to motivate the patient for treatment rigors and future effective utilisation of denture. The slow raising of the dental chair for upright position is recommended to prevent the orthostatic hypotension [11].

The success of the complete denture is predominantly dependent on the neuromuscular control of the oral musculature. The mandibular movement is moderated by the feedback from the periodontal ligament, temporomandibular joint, and musculature receptors. The edentulous PD patients seriously compromised in this feedback; hence implant or tooth-supported over dentures are advantageous for better proprioception and controlled jaw movement [12, 13]. The involuntary muscle movement, xerostomia, and rigid musculature in PD patients compromise the denture retention and control. It is advantageous to use the quick setting impression material in severe form of PD. The low fusing impression compound was used for border moulding due to its incremental procedure. It provides a chance for subsequent correction and helping the patient to concentrate on one muscular movement at a time. The semireclined 45-degree position during impression procedure is advantageous for avoiding excessive saliva pooling and avoiding the risk of choking [11]. Since the patient was unable to perform the functional mandibular movement, we were unable to apply the Gothic arch tracing method to record the horizontal relation. Instead, the bilateral manipulation technique was utilized to guide the mandible to centric relation [14]. The neutral zone teeth arrangement was observed to enhance the denture stability and retention. The researchers observe that the teeth at neutral zone do not interfere with involuntary muscular movement in PD patient [15]. The cusp-less teeth are indicated for the patients with poor muscular control to accommodate irregular mandibular movement. The lingualized occlusion scheme was utilized in the patient due to its combined advantage of better masticatory efficiency [16, 17], less distortion, and limited lateral movement of denture [18, 19]. The researchers have also reported that lingualized occlusion is suitable for the patient with involuntary teeth grinding [20]. The relatively flat lower teeth are hypothesized to increase the feedback from masseter muscle and mucosa, hence helping the patient in improved proprioception and smooth mandibular movement. The burning mouth syndrome is common in PD patient due to xerostomia; it is more aggravated by denture wearing [21]. Frequent water sipping and artificial saliva substitute are helpful in these patients. Water based denture adhesive and denture cleansers will also help in improving the denture wearing confidence and denture hygiene. Posttreatment follow-up is critical for successful rehabilitation. It is helpful for continuous monitoring, evaluation, and correction of denture. The effective complete denture rehabilitation will help PD patient in alleviating both psychological and physical debilities to significant extent [22].

## 4. Conclusion

The significant number of PD patients in society requires the complete denture for the functional, aesthetic, and psychological rehabilitation. Along with caring, sympathetic approach, treatment plan should include a strategy to overcome the physical disabilities of the patient. Patient motivation, education, and posttreatment follow-up are critical for the successful treatment outcome with complete denture.
